# Supplementary material for: Pyruvate Dehydrogenase Kinase 1 inhibition mediated oxidative phosphorylation enhancement in cartilage promotes osteoarthritis progression
Source: BMC Musculoskelet Disord. 2023 Jul 20;24:597. doi: 10.1186/s12891-023-06585-6 (PMC10357736; doi:10.1186/s12891-023-06585-6)
Supplement: Supplementary file 1 — Additional file 1. [file 12891_2023_6585_MOESM1_ESM.zip › KEGG.Gsea.1653622432464/KEGG_ANTIGEN_PROCESSING_AND_PRESENTATION.html]

Details for gene set KEGG\_ANTIGEN\_PROCESSING\_AND\_PRESENTATION[GSEA]

|  || Dataset | 55457\_collapsed\_to\_symbols.cls.cls #OA\_versus\_normal.cls.cls #OA\_versus\_normal\_repos |
| Phenotype | cls.cls#OA\_versus\_normal\_repos |
| Upregulated in class | normal |
| GeneSet | KEGG\_ANTIGEN\_PROCESSING\_AND\_PRESENTATION |
| Enrichment Score (ES) | -0.26380032 |
| Normalized Enrichment Score (NES) | -1.1036463 |
| Nominal p-value | 0.27422002 |
| FDR q-value | 0.5379565 |
| FWER p-Value | 1.0 |
Table: GSEA Results Summary

  

Fig 1: Enrichment plot: KEGG\_ANTIGEN\_PROCESSING\_AND\_PRESENTATION      
 Profile of the Running ES Score & Positions of GeneSet Members on the Rank Ordered List

  

| SYMBOL | TITLE | RANK IN GENE LIST | RANK METRIC SCORE | RUNNING ES | CORE ENRICHMENT || 1 | HSPA2 | heat shock protein family A (Hsp70) member 2 [Source:HGNC Symbol;Acc:HGNC:5235] | 662 | 0.603 | -0.0226 | No |
| 2 | HLA-DMB | "major histocompatibility complex, class II, DM beta [Source:HGNC Symbol;Acc:HGNC:4935]" | 679 | 0.598 | 0.0050 | No |
| 3 | NFYC | nuclear transcription factor Y subunit gamma [Source:HGNC Symbol;Acc:HGNC:7806] | 1104 | 0.495 | -0.0042 | No |
| 4 | HSPA1L | heat shock protein family A (Hsp70) member 1 like [Source:HGNC Symbol;Acc:HGNC:5234] | 1139 | 0.488 | 0.0167 | No |
| 5 | IFNA8 | interferon alpha 8 [Source:HGNC Symbol;Acc:HGNC:5429] | 1206 | 0.475 | 0.0345 | No |
| 6 | HLA-DQA1 | "major histocompatibility complex, class II, DQ alpha 1 [Source:HGNC Symbol;Acc:HGNC:4942]" | 1334 | 0.452 | 0.0463 | No |
| 7 | RFX5 | regulatory factor X5 [Source:HGNC Symbol;Acc:HGNC:9986] | 1343 | 0.451 | 0.0675 | No |
| 8 | RFXANK | regulatory factor X associated ankyrin containing protein [Source:HGNC Symbol;Acc:HGNC:9987] | 1383 | 0.445 | 0.0859 | No |
| 9 | HLA-DMA | "major histocompatibility complex, class II, DM alpha [Source:HGNC Symbol;Acc:HGNC:4934]" | 1769 | 0.388 | 0.0746 | No |
| 10 | HLA-DOB | "major histocompatibility complex, class II, DO beta [Source:HGNC Symbol;Acc:HGNC:4937]" | 2147 | 0.344 | 0.0618 | No |
| 11 | CTSS | cathepsin S [Source:HGNC Symbol;Acc:HGNC:2545] | 2280 | 0.329 | 0.0674 | No |
| 12 | HLA-DQB1 | "major histocompatibility complex, class II, DQ beta 1 [Source:HGNC Symbol;Acc:HGNC:4944]" | 2840 | 0.266 | 0.0366 | No |
| 13 | KLRC3 | killer cell lectin like receptor C3 [Source:HGNC Symbol;Acc:HGNC:6376] | 3243 | 0.227 | 0.0162 | No |
| 14 | PSME1 | proteasome activator subunit 1 [Source:HGNC Symbol;Acc:HGNC:9568] | 3388 | 0.213 | 0.0152 | No |
| 15 | KLRC4 | killer cell lectin like receptor C4 [Source:HGNC Symbol;Acc:HGNC:6377] | 3563 | 0.197 | 0.0111 | No |
| 16 | RFXAP | regulatory factor X associated protein [Source:HGNC Symbol;Acc:HGNC:9988] | 3624 | 0.191 | 0.0157 | No |
| 17 | CD8B | CD8b molecule [Source:HGNC Symbol;Acc:HGNC:1707] | 4226 | 0.135 | -0.0247 | No |
| 18 | IFNA4 | interferon alpha 4 [Source:HGNC Symbol;Acc:HGNC:5425] | 4282 | 0.129 | -0.0227 | No |
| 19 | HLA-DOA | "major histocompatibility complex, class II, DO alpha [Source:HGNC Symbol;Acc:HGNC:4936]" | 4344 | 0.125 | -0.0215 | No |
| 20 | NFYB | nuclear transcription factor Y subunit beta [Source:HGNC Symbol;Acc:HGNC:7805] | 4389 | 0.122 | -0.0190 | No |
| 21 | CD4 | CD4 molecule [Source:HGNC Symbol;Acc:HGNC:1678] | 4465 | 0.116 | -0.0193 | No |
| 22 | TAPBP | TAP binding protein [Source:HGNC Symbol;Acc:HGNC:11566] | 4527 | 0.111 | -0.0187 | No |
| 23 | PSME2 | proteasome activator subunit 2 [Source:HGNC Symbol;Acc:HGNC:9569] | 4672 | 0.098 | -0.0252 | No |
| 24 | KLRD1 | killer cell lectin like receptor D1 [Source:HGNC Symbol;Acc:HGNC:6378] | 4778 | 0.089 | -0.0291 | No |
| 25 | LGMN | legumain [Source:HGNC Symbol;Acc:HGNC:9472] | 5433 | 0.038 | -0.0783 | No |
| 26 | IFI30 | IFI30 lysosomal thiol reductase [Source:HGNC Symbol;Acc:HGNC:5398] | 5483 | 0.033 | -0.0805 | No |
| 27 | HLA-DPB1 | "major histocompatibility complex, class II, DP beta 1 [Source:HGNC Symbol;Acc:HGNC:4940]" | 5486 | 0.033 | -0.0790 | No |
| 28 | IFNA2 | interferon alpha 2 [Source:HGNC Symbol;Acc:HGNC:5423] | 5869 | 0.004 | -0.1086 | No |
| 29 | KIR3DL2 | "killer cell immunoglobulin like receptor, three Ig domains and long cytoplasmic tail 2 [Source:HGNC Symbol;Acc:HGNC:6339]" | 5893 | 0.002 | -0.1103 | No |
| 30 | IFNA21 | interferon alpha 21 [Source:HGNC Symbol;Acc:HGNC:5424] | 6127 | -0.015 | -0.1277 | No |
| 31 | CD74 | CD74 molecule [Source:HGNC Symbol;Acc:HGNC:1697] | 6374 | -0.033 | -0.1453 | No |
| 32 | HLA-DRA | "major histocompatibility complex, class II, DR alpha [Source:HGNC Symbol;Acc:HGNC:4947]" | 6550 | -0.046 | -0.1568 | No |
| 33 | CD8A | CD8a molecule [Source:HGNC Symbol;Acc:HGNC:1706] | 6579 | -0.048 | -0.1566 | No |
| 34 | PDIA3 | protein disulfide isomerase family A member 3 [Source:HGNC Symbol;Acc:HGNC:4606] | 6607 | -0.050 | -0.1563 | No |
| 35 | TAP1 | "transporter 1, ATP binding cassette subfamily B member [Source:HGNC Symbol;Acc:HGNC:43]" | 6633 | -0.052 | -0.1558 | No |
| 36 | IFNA10 | interferon alpha 10 [Source:HGNC Symbol;Acc:HGNC:5418] | 6682 | -0.054 | -0.1569 | No |
| 37 | IFNA5 | interferon alpha 5 [Source:HGNC Symbol;Acc:HGNC:5426] | 6755 | -0.059 | -0.1596 | No |
| 38 | KLRC1 | killer cell lectin like receptor C1 [Source:HGNC Symbol;Acc:HGNC:6374] | 6835 | -0.066 | -0.1626 | No |
| 39 | PSME3 | proteasome activator subunit 3 [Source:HGNC Symbol;Acc:HGNC:9570] | 6903 | -0.072 | -0.1644 | No |
| 40 | HLA-DRB1 | "major histocompatibility complex, class II, DR beta 1 [Source:HGNC Symbol;Acc:HGNC:4948]" | 7087 | -0.086 | -0.1745 | No |
| 41 | HLA-F | "major histocompatibility complex, class I, F [Source:HGNC Symbol;Acc:HGNC:4963]" | 7196 | -0.096 | -0.1783 | No |
| 42 | IFNA6 | interferon alpha 6 [Source:HGNC Symbol;Acc:HGNC:5427] | 7272 | -0.101 | -0.1793 | No |
| 43 | CALR | calreticulin [Source:HGNC Symbol;Acc:HGNC:1455] | 7291 | -0.102 | -0.1758 | No |
| 44 | KIR2DL4 | "killer cell immunoglobulin like receptor, two Ig domains and long cytoplasmic tail 4 [Source:HGNC Symbol;Acc:HGNC:6332]" | 7384 | -0.109 | -0.1777 | No |
| 45 | CREB1 | cAMP responsive element binding protein 1 [Source:HGNC Symbol;Acc:HGNC:2345] | 7470 | -0.116 | -0.1787 | No |
| 46 | CANX | calnexin [Source:HGNC Symbol;Acc:HGNC:1473] | 7672 | -0.132 | -0.1880 | No |
| 47 | HSPA1A | heat shock protein family A (Hsp70) member 1A [Source:HGNC Symbol;Acc:HGNC:5232] | 7876 | -0.146 | -0.1968 | No |
| 48 | KIR2DL1 | "killer cell immunoglobulin like receptor, two Ig domains and long cytoplasmic tail 1 [Source:HGNC Symbol;Acc:HGNC:6329]" | 8348 | -0.182 | -0.2247 | No |
| 49 | KIR2DS4 | "killer cell immunoglobulin like receptor, two Ig domains and short cytoplasmic tail 4 [Source:HGNC Symbol;Acc:HGNC:6336]" | 8519 | -0.195 | -0.2286 | No |
| 50 | IFNA7 | interferon alpha 7 [Source:HGNC Symbol;Acc:HGNC:5428] | 8533 | -0.196 | -0.2201 | No |
| 51 | IFNA1 | interferon alpha 1 [Source:HGNC Symbol;Acc:HGNC:5417] | 8640 | -0.204 | -0.2186 | No |
| 52 | HSPA8 | heat shock protein family A (Hsp70) member 8 [Source:HGNC Symbol;Acc:HGNC:5241] | 9140 | -0.243 | -0.2458 | No |
| 53 | CIITA | class II major histocompatibility complex transactivator [Source:HGNC Symbol;Acc:HGNC:7067] | 9184 | -0.246 | -0.2373 | No |
| 54 | HLA-DPA1 | "major histocompatibility complex, class II, DP alpha 1 [Source:HGNC Symbol;Acc:HGNC:4938]" | 9481 | -0.273 | -0.2472 | No |
| 55 | KIR2DL5A | "killer cell immunoglobulin like receptor, two Ig domains and long cytoplasmic tail 5A [Source:HGNC Symbol;Acc:HGNC:16345]" | 9695 | -0.291 | -0.2498 | Yes |
| 56 | HSPA4 | heat shock protein family A (Hsp70) member 4 [Source:HGNC Symbol;Acc:HGNC:5237] | 9797 | -0.301 | -0.2431 | Yes |
| 57 | LTA | lymphotoxin alpha [Source:HGNC Symbol;Acc:HGNC:6709] | 9921 | -0.313 | -0.2376 | Yes |
| 58 | HSP90AB1 | heat shock protein 90 alpha family class B member 1 [Source:HGNC Symbol;Acc:HGNC:5258] | 10060 | -0.327 | -0.2326 | Yes |
| 59 | NFYA | nuclear transcription factor Y subunit alpha [Source:HGNC Symbol;Acc:HGNC:7804] | 10297 | -0.350 | -0.2341 | Yes |
| 60 | CTSB | cathepsin B [Source:HGNC Symbol;Acc:HGNC:2527] | 10313 | -0.352 | -0.2183 | Yes |
| 61 | HSPA6 | heat shock protein family A (Hsp70) member 6 [Source:HGNC Symbol;Acc:HGNC:5239] | 10561 | -0.379 | -0.2193 | Yes |
| 62 | KIR3DL3 | "killer cell immunoglobulin like receptor, three Ig domains and long cytoplasmic tail 3 [Source:HGNC Symbol;Acc:HGNC:16312]" | 10678 | -0.390 | -0.2095 | Yes |
| 63 | CTSL | cathepsin L [Source:HGNC Symbol;Acc:HGNC:2537] | 10817 | -0.407 | -0.2007 | Yes |
| 64 | HSPA5 | heat shock protein family A (Hsp70) member 5 [Source:HGNC Symbol;Acc:HGNC:5238] | 10820 | -0.407 | -0.1812 | Yes |
| 65 | HLA-G | "major histocompatibility complex, class I, G [Source:HGNC Symbol;Acc:HGNC:4964]" | 10869 | -0.413 | -0.1650 | Yes |
| 66 | KIR2DS3 | "killer cell immunoglobulin like receptor, two Ig domains and short cytoplasmic tail 3 [Source:HGNC Symbol;Acc:HGNC:6335]" | 11103 | -0.444 | -0.1618 | Yes |
| 67 | TAP2 | "transporter 2, ATP binding cassette subfamily B member [Source:HGNC Symbol;Acc:HGNC:44]" | 11616 | -0.519 | -0.1767 | Yes |
| 68 | HLA-E | "major histocompatibility complex, class I, E [Source:HGNC Symbol;Acc:HGNC:4962]" | 11659 | -0.526 | -0.1546 | Yes |
| 69 | KIR3DL1 | "killer cell immunoglobulin like receptor, three Ig domains and long cytoplasmic tail 1 [Source:HGNC Symbol;Acc:HGNC:6338]" | 11864 | -0.563 | -0.1434 | Yes |
| 70 | HSPA1B | heat shock protein family A (Hsp70) member 1B [Source:HGNC Symbol;Acc:HGNC:5233] | 12278 | -0.670 | -0.1433 | Yes |
| 71 | HLA-A | "major histocompatibility complex, class I, A [Source:HGNC Symbol;Acc:HGNC:4931]" | 12502 | -0.749 | -0.1246 | Yes |
| 72 | B2M | beta-2-microglobulin [Source:HGNC Symbol;Acc:HGNC:914] | 12507 | -0.750 | -0.0888 | Yes |
| 73 | HLA-B | "major histocompatibility complex, class I, B [Source:HGNC Symbol;Acc:HGNC:4932]" | 12534 | -0.764 | -0.0539 | Yes |
| 74 | HLA-DRB4 | "major histocompatibility complex, class II, DR beta 4 [Source:HGNC Symbol;Acc:HGNC:4952]" | 12636 | -0.815 | -0.0225 | Yes |
| 75 | HSP90AA1 | heat shock protein 90 alpha family class A member 1 [Source:HGNC Symbol;Acc:HGNC:5253] | 12730 | -0.893 | 0.0133 | Yes |
Table: GSEA details [plain text format]

  

Fig 2: KEGG\_ANTIGEN\_PROCESSING\_AND\_PRESENTATION      
 Blue-Pink O' Gram in the Space of the Analyzed GeneSet

  

Fig 3: KEGG\_ANTIGEN\_PROCESSING\_AND\_PRESENTATION: Random ES distribution      
 Gene set null distribution of ES for **KEGG\_ANTIGEN\_PROCESSING\_AND\_PRESENTATION**
